# Outer Membrane Vesicles Derived from Adherent-Invasive *Escherichia coli* Induce Inflammatory Response and Alter the Gene Expression of Junction-Associated Proteins in Human Intestinal Epithelial Cells

**DOI:** 10.1155/2024/2701675

**Published:** 2024-05-24

**Authors:** Bahareh Nadalian, Banafsheh Nadalian, Mohammad Reza Zali, Abbas Yadegar

**Affiliations:** ^1^Foodborne and Waterborne Diseases Research Center, Research Institute for Gastroenterology and Liver Diseases, Shahid Beheshti University of Medical Sciences, Tehran, Iran; ^2^Gastroenterology and Liver Diseases Research Center, Research Institute for Gastroenterology and Liver Diseases, Shahid Beheshti University of Medical Sciences, Tehran, Iran; ^3^Basic and Molecular Epidemiology of Gastrointestinal Disorders Research Center, Research Institute for Gastroenterology and Liver Diseases, Shahid Beheshti University of Medical Sciences, Tehran, Iran

## Abstract

Adherent-invasive *Escherichia coli* (AIEC) pathobionts, which are characterized by their ability to adhere to and invade intestinal epithelial cells, are associated with the etiopathogenesis of inflammatory bowel diseases (IBDs). Outer membrane vesicles (OMVs) released by AIEC strains can facilitate the interaction of these bacteria with host cells through delivering bacterial effectors. The aim of this study was to determine the ability of OMVs derived from AIEC strain LF82 to induce the host immune response, leading to production of proinflammatory cytokines and also altering the gene expression of junction-associated proteins in the human epithelial colorectal adenocarcinoma Caco-2 cell line. OMVs were extracted from AIEC strain LF82, and the cell viability of Caco-2 cells treated with these vesicles was assessed by MTT assay. The morphology and size distribution of vesicles were analyzed using transmission electron microscopy and dynamic light scattering, respectively. Gene expression of *occludin*, *ZO-1*, *claudin-2*, *E-cadherin*, TLR-2, and TLR-4 in response to OMVs was assessed in Caco-2 cells by RT-qPCR. Moreover, the secretion of IL-8 and TNF*-α* into the supernatant of Caco-2 cells upon treatment with OMVs was measured using ELISA. Our results demonstrated that OMVs upregulated the gene expression level of TLRs and also altered the gene expression level of junction-associated proteins. OMVs derived from AIEC may play a major role in the promotion of intestinal inflammation and epithelial barrier dysfunction. However, further investigations are needed to elucidate the putative role of OMVs in the pathogenesis of AIEC and IBD.

## 1. Introduction

The human gut microbiota that is made up of a complex and dynamic microbial community provides the host with several functions, such as nutrient availability and energy harvest, protection against pathogens, and development of metabolism and immune system [1]. These indigenous microorganisms also can modulate the paracellular barrier permeability in the intestinal epithelium through the regulation of tight junctions leading to intestinal homeostasis [2]. Recent evidence indicated that microbial imbalances, also known as gut dysbiosis, may result in impaired intestinal barrier integrity and gut barrier dysfunction, which can dysregulate the host immune response and increase the risk of inflammatory diseases [3, 4]. Although the vast majority of the intestinal microbial communities are mutualistic or commensals, some resident symbionts that are often referred to as pathobionts can promote pathology under specific host genetic predisposition and compromised immune status [5]. In contrast to opportunistic pathogens, which are described to be involved in acute infections, pathobionts can affect the host indirectly via induction of the immune system and are often associated with chronic inflammatory conditions [6]. *Escherichia coli*, classified as a member of the family Enterobacteriaceae, is a facultative anaerobic Gram-negative bacterium, which is one of the most prevalent species of the normal human gut [7, 8]. This gut microbe can promote the stability of the gut microbiota and helps to maintain human intestinal homeostasis. Although *E. coli* bacteria are defined as common inhabitants of the gastrointestinal tract, several *E. coli* strains have been able to acquire a virulent nature [7]. A specific *E. coli* pathotype named adherent‐invasive *E. coli* (AIEC) is suspected to be implicated in the etiopathogenesis of inflammatory bowel diseases (IBD), including Crohn disease (CD) and ulcerative colitis (UC) [9, 10]. AIEC strains are considered to be pathobionts that can trigger intestinal inflammation in susceptible hosts due to their genome evolution and adaptation [6–8]. The prevalence of AIEC has been found to be increased in both CD and UC compared to healthy individuals [9]. Although typical virulence factors related to pathogenic *E. coli* have not been found in AIEC, these strains harbor several adhesive and invasive properties, as well as the ability to survive and replicate within macrophage [11].

Gram-negative bacteria release vesicles known as outer membrane vesicles (OMVs) under various growth conditions. OMVs are spherical bilayer nanostructures ranging in size from 20 to 250 nm, which transport bacterial effectors into host cells [12]. These vesicles carry out diverse biological functions and play important roles in several processes, such as intracellular and extracellular communication, nutrient acquisition, biofilm formation, virulence and pathogenesis, modulation of the host immune response, and horizontal gene transfer [12, 13]. Because of the particulate content and innate composition of OMVs, these nanoscale particles represent a complex array of pathogen-associated molecular patterns (PAMPs) that can be recognized by the immune system [14]. The interaction of OMV-associated PAMPs with pattern recognition receptors (PRRs), which are ubiquitously expressed by innate immune and epithelial cells, can result in the induction of various signaling cascades, thereby activating the host immune response [12, 14, 15]. Toll-like receptors (TLRs), a family of PRRs, are key components of the innate immune system that regulate inflammatory reactions [15]. Due to the putative role of AIEC strains in promoting inflammatory diseases and given the high proportion of PAMPs on OMVs, we hypothesized that OMVs produced by these pathobionts may provoke the host immune response and disrupt the integrity of intestinal epithelial cells. The present study was conducted to investigate the production of inflammatory cytokines in the human epithelial colorectal adenocarcinoma Caco-2 cell line upon coculture with OMVs derived from AIEC strain LF82. Quantitative real-time PCR (RT-qPCR) analysis was also performed to assess the effects of AIEC-derived OMVs on gene expression of tight junction and adherens junction proteins *in vitro*.

## 2. Materials and Methods

### 2.1. Bacterial Strains and Growth Conditions

Two *E. coli* strains were used in this study, including nonpathogenic *E. coli* strain DH5*α* and AIEC reference strain LF82 (kindly was gifted from Université Clermont Auvergne, France). The bacterial strains were directly cultured on the Luria–Bertani (LB) agar plate and aerobically grown overnight at 37°C. A single colony from the LB agar plate was used to inoculate LB broth and incubated overnight at 37°C for further OMV isolation.

### 2.2. Preparation of OMVs

OMVs from AIEC strain LF82 were isolated as previously described with some minor modifications [16]. Briefly, bacterial pellets were harvested by centrifugation at 10, 000 × *g* and the supernatants were centrifuged further to remove any remaining bacterial cells and cell debris. The obtained supernatants were filtered through a 0.20 *µ*m pore-size (Sigma-Aldrich, St. Louis, MO, USA) filter and extracted by ultracentrifugation at 150, 000 × *g* for 3 h at 4°C. The pellets containing OMVs were resuspended in sterile phosphate-buffered saline (PBS, pH = 7) and stored at −80°C until use. The total protein concentration in the purified OMVs was determined using the bicinchoninic acid (BCA) protein assay (DNAbiotech, Tehran, Iran).

### 2.3. Sodium Dodecyl Sulfate-Polyacrylamide Gel Electrophoresis

The protein profile of OMVs and whole cell lysate were analyzed by SDS-PAGE. The extracted OMVs were resuspended in SDS-PAGE loading buffer and heated at 95°C for 5 min. To prepare whole cell lysate, bacterial cells were pelleted and resuspended in SDS-PAGE loading buffer, and then heated at 95°C for 10 min. Afterward, the protein samples from OMVs and whole cell lysate were loaded onto a 12% polyacrylamide gel, followed by gel staining with Coomassie Brilliant Blue as previously described [17].

### 2.4. Transmission Electron Microscopy and Dynamic Light Scattering

TEM analysis was applied to assess the size and structure of the isolated OMVs. In brief, OMVs were diluted using PBS, and 10 *μ*L of suspension was placed on a 400-mesh copper grid with carbon-coated formvar film. The grid was then stained with 2% (w/v) uranyl acetate, and after blotting and repeating, the grid became air-dried. Eventually, the images were captured using a TEM microscope (Carl Zeiss, Oberkochen, Germany). The size distribution of the extracted OMVs from AIEC strain LF82 was determined by DLS using a Zetasizer Nano ZS (ZEN3600, Malvern Instruments, UK).

### 2.5. Cell Culture

The human colorectal adenocarcinoma Caco-2 cell line (ATCC® HTB-37™) was obtained from the Iranian Biological Resource Center, Tehran, Iran. The cells were routinely maintained in high-glucose Dulbecco's Modified Eagle Medium (H-DMEM, Gibco, USA) supplemented with 1% 100 U/mL of penicillin, and 100 *μ*g/mL of streptomycin and 10% heat-inactivated fetal bovine serum (FBS) (Gibco-Invitrogen, Carlsbad, CA) in a 5% CO_2_ humidified incubator at 37°C.

### 2.6. Cell Viability Assay

The viability of Caco-2 cells was assessed using the MTT assay (Sigma-Aldrich, St. Louis, MO, USA). Briefly, 5 × 10^4^ cells/well were seeded in 96-well plates and incubated at 37°C. Afterward, monolayer cells were treated separately with live LF82 and DH5*α* strains at two levels of multiplicity of infection (MOI) of 10 (≈1 × 10^7^ CFU/mL) and 100 (≈1 × 10^8^ CFU/mL), lipopolysaccharide (LPS) from *E. coli* O111:B4 (Sigma-Aldrich, St. Louis, MO, USA) at various concentrations (0.1, 1, and 10 *µ*g/mL), and different concentrations of OMVs (0.1, 1, 10, and 50 *µ*g/mL) for 3, 6, 12, and 24 h time points. After incubation, 10 *μ*L of 3-(4,5-dimethylthiazol-2-yl)-2,5-diphenyltetrazolium bromide (MTT) reagent was added to each well, and the plate was incubated for 4 h at 37°C. The media was then discarded, and 200 *μ*L of dimethyl sulfoxide (DMSO) was added to each well to stop the assay. Finally, the absorbance of the wells was read at 570 nm with correction at 630 nm using a microplate reader (BioTek, USA). The percentage of cell viability was calculated using the following formula:

(1) $$\begin{array}{c} \text{Cell viability }\left(\%\right)=\frac{\left(X\times 100\%\right)}{Y} \end{array}$$

where “*X*” is the absorbance of treated cells and “*Y*” is the absorbance of untreated cells.

### 2.7. Cell Treatments

Caco-2 cells were seeded at 10^5^ cells/well in a 24-well plate and grown in a CO_2_ incubator at 37°C to reach a confluence of 80–90%. The culture medium of Caco-2 cell monolayers was replaced with antibiotic/serum-free complete DMEM overnight. Then, the cells were infected separately with live LF82 and DH5*α*, as control strains, at a MOI 10 and OMVs at a concentration of 10 *µ*g/mL for 24 h in the CO_2_ incubator at 37°C. Untreated cells and cells treated with PBS were served as the control groups for the same period of time. Each experiment was performed in duplicate and repeated at least three times. In separate experiments, OMVs, and live LF82 and DH5*α* strains were pretreated with 10 *µ*g/mL polymyxin B (Himedia, Mumbai, India) for 1 h to neutralize any action of LPS, and then, Caco-2 cells were incubated with either of these polymyxin B-pretreated samples. Also, LPS (0.1 *µ*g/mL) was used as a control in these experiments.

### 2.8. RNA Extraction and cDNA Synthesis

Total RNA was extracted from Caco-2 cells using RNeasy Plus Mini Kit (Qiagen, Germany) as per the manufacturer's instruction. RNA concentration and purity were assessed spectrophotometrically via NanoDrop spectrophotometer (ND-1000, Thermo Scientific, USA) and visually using agarose gel electrophoresis. cDNA was synthesized by reverse transcription of the purified RNA using BioFACT RT-kit (BIOFACT, South Korea) according to the supplier's protocol.

### 2.9. Gene Expression Analysis by RT-qPCR

Gene expression analysis of the target genes was performed by RT-qPCR amplification using Rotor-Gene Q (Qiagen, Germany) real-time PCR instrument using BioFACT 2× Real-Time PCR Master Mix (BIOFACT, South Korea). The list of target genes and oligonucleotide primers used for real-time PCR assays is presented in Table 1. The relative gene expression of *occludin*, *ZO-1*, *claudin-2*, *E-cadherin*, TLR-4, and TLR-2 was determined using the 2^−ΔΔCt^ method. Housekeeping gene *β-*actin was used as an internal control to normalize the gene expression level.

### 2.10. Measurement of Cytokines by ELISA

Following treatment, cell culture supernatants were collected and maintained at −20°C until use. The level of interleukin 8 (IL-8) and tumor necrosis factor-alpha (TNF-*α*) was measured in cell culture supernatants by using the enzyme-linked immunosorbent assay (ELISA) kit (ZellBio GmbH, Germany) according to the manufacturer's instruction.

### 2.11. Statistical Analysis

Statistical analysis was carried out with GraphPad Prism software version 8 (GraphPad Software, Inc., CA, USA). One-way analysis of variance (ANOVA) followed by Tukey's post hoc test (for multiple comparisons between more than two groups) was used to calculate statistical significance between groups. Results are presented as the average ± standard error of mean (SEM) of at least three experiments, unless otherwise stated. Differences were considered statistically significant when *P* < 0.05, ^*∗*^*P* < 0.05, ^*∗∗*^*P* < 0.01, ^*∗∗∗*^*P* < 0.001, and ^*∗∗∗∗*^*P* < 0.0001.

## 3. Results

### 3.1. Characterization and Morphology of OMVs Isolated from AIEC Strain LF82

**Figure 1:**
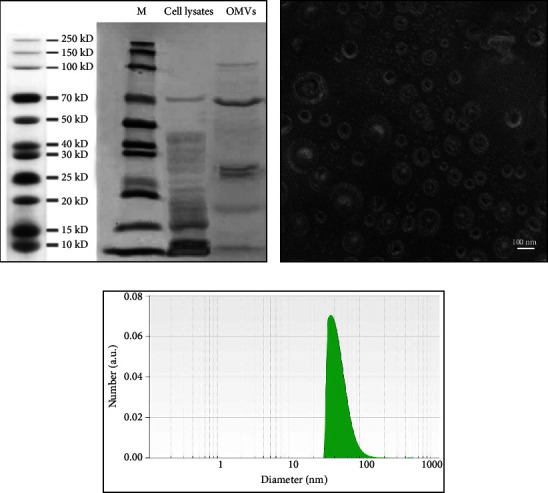
Characterization of the OMVs derived from AIEC strain LF82. (a) Analysis of the protein contents of whole cell lysate and the OMVs separated by 12% SDS-PAGE followed by visualization of proteins using Coomassie blue staining. Lines to the left indicate the molecular masses of the protein ladder in kDa. (b) Representative transmission electron microscopy of the OMVs isolated from AIEC strain LF82. Scale bar (lower right) = 100 nm. (c) Dynamic light scattering indicates the size distribution of the isolated OMVs.

OMVs were successfully isolated from the culture supernatant of AIEC strain LF82, and SDS-PAGE was performed to identify the protein profile of the vesicles. As shown in Figure 1(a), OMVs contain distinct protein profile compared to whole cell lysate. The structure and morphological characteristics of the purified OMVs were assessed by TEM. The results revealed that LF82-derived OMVs are spherical and bilayered nanosized particles (Figure 1(b)). To further characterize the OMVs, their size distribution was determined using DLS analysis. The majority of the vesicles ranged in size between 40 and 100 nm (Figure 1(c)).

### 3.2. Cell Viability of Caco-2 Cells Treated with LF82-Derived OMVs

**Figure 2:**
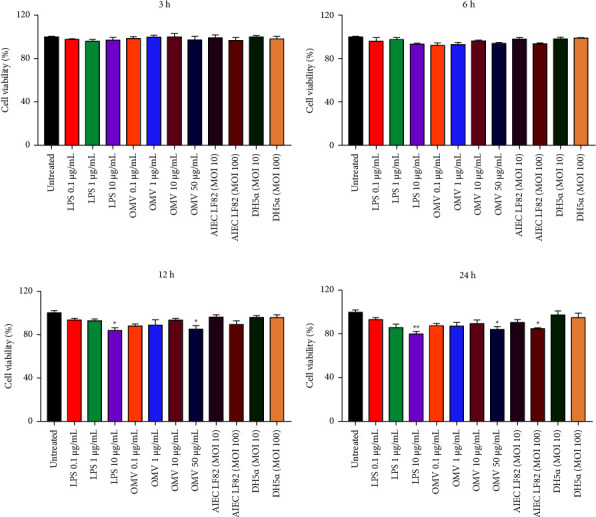
Cell viability of Caco-2 cells upon treatment with different concentrations of LPS (0.1, 1, 10 *µ*g/mL), varying concentrations of AIEC OMVs (0.1, 1, 10, 50 *µ*g/mL), and the live strains of LF82 and DH5*α* (MOIs 10 and 100) for (a) 3 h, (b) 6 h, (c) 12 h, and (d) 24 h time points. Data were shown as the mean ± SEM. ^*∗*^*P* < 0.05; ^*∗∗*^*P* < 0.01; ^*∗∗∗*^*P* < 0.001; ^*∗∗∗∗*^*P* < 0.0001 by post hoc Tukey's one-way ANOVA statistical analysis.

MTT assay was performed to determine the cell viability of Caco-2 cells after exposure to LF82-derived OMVs, live LF82, LPS, and DH5*α* for 3, 6, 12, and 24 h time points and compared to untreated cells (Figures 2(a)–2(d)). Based on the MTT assay results, the LF82 strain at MOI 10 and DH5*α* strain at both MOIs (10 and 100) did not induce a significant increase or decrease in the number of viable Caco-2 cells during all time points, while LF82 strain at MOI 100 significantly (*P* < 0.05) reduced the cell viability of Caco-2 cells after 24 h of treatment. Furthermore, MTT assay demonstrated that LF82-derived OMVs at concentration of 50 *µ*g/mL significantly (*P* < 0.05) decreased the cell viability of Caco-2 cells after 24 h compared with untreated cells. In addition, LPS treatment at concentration of 10 *µ*g/mL significantly affected the cell viability of Caco-2 cells compared with untreated cells after 12 h (*P* < 0.05) and 24 h (*P* < 0.01). Similarly, OMVs at concentration of 50 *µ*g/mL caused a significant reduction in the viability of Caco-2 cells after 12 h (*P* < 0.05). No significant effect was observed on viability of Caco-2 cells after 3 and 6 h of treatment with OMVs and LPS at different concentrations in comparison with untreated cells.

### 3.3. OMVs Derived from AIEC Strain LF82 Induced the Gene Expression of TLR-2 and TLR-4

**Figure 3:**
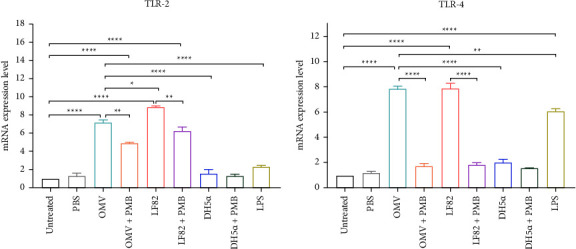
Gene expression of (a) TLR-2 and (b) TLR-4 in Caco-2 cells treated with AIEC OMVs, and the live strains of LF82 and DH5*α*. *β-actin* served as the reference gene for the normalization of data in RT-qPCR experiments. Data were expressed as the mean ± SEM. ^*∗*^*P* < 0.05; ^*∗∗*^*P* < 0.01; ^*∗∗∗*^*P* < 0.001; ^*∗∗∗∗*^*P* < 0.0001 by post hoc Tukey's one-way ANOVA statistical analysis.

To evaluate the effects of LF82-derived OMVs on gene expression level of TLR-2 and TLR-4, Caco-2 cells were treated with OMVs at concentration of 10 *µ*g/mL for 24 h. The effects of live LF82 and DH5*α*, as control strains, on the gene expression of TLR-2 and TLR-4 were also compared with that of OMVs (Figures 3(a) and 3(b)). The results of RT-qPCR assays demonstrated that the expression of TLR-2 and TLR-4 significantly increased in Caco-2 cells treated with OMVs compared to untreated cells (*P* < 0.0001). In addition, there was a significant difference between the impact of live LF82 and its derived OMVs on gene expression of TLR-2, in which OMVs showed a smaller effect (*P* < 0.05). However, live LF82 and its derived OMVs had an almost similar effect on the gene expression level of TLR-4. Caco-2 cells treated with OMVs showed higher mRNA expression level of TLR-2 and TLR-4 compared to cells treated with DH5*α* (*P* < 0.0001). Of note, LF82 OMVs had a greater impact on the induction of the mRNA expression of TLR-4 in Caco-2 cells when compared to that of LPS (*P* < 0.01). Moreover, to abolish the effect of LPS on the gene expression of TLR-2 and TLR-4, OMVs and live bacteria (LF82 and DH5*α* strains) were pretreated with polymyxin B and added to Caco-2 cells. Gene expression analysis demonstrated that polymyxin B pretreatment reduced the ability of OMVs and live LF82 to induce the gene expression of TLR-2 and TLR-4 in Caco-2 cells.

### 3.4. OMVs Isolated from AIEC Strain LF82 Altered the Gene Expression of Junction-Associated Proteins

**Figure 4:**
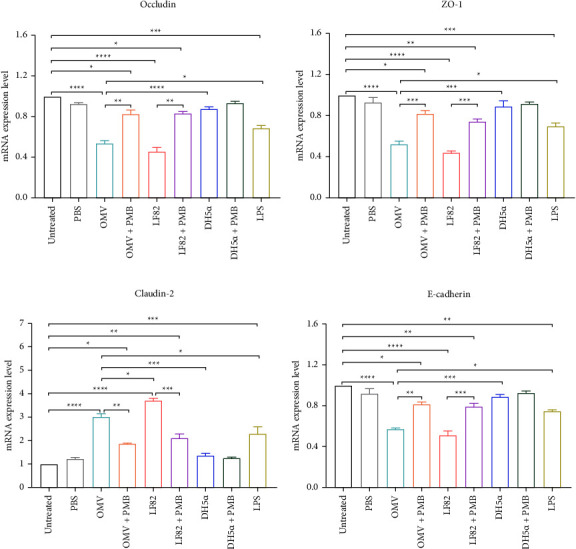
Gene expression of (a) *occludin*, (b) *ZO-1*, (c) *claudin-2*, and (d) *E-cadherin* in Caco-2 cells treated with AIEC OMVs, and the live strains of LF82 and DH5*α*. *β-*actin served as the reference gene for the normalization of data in RT-qPCR experiments. Data were expressed as the mean ± SEM. ^*∗*^*P* < 0.05; ^*∗∗*^*P* < 0.01; ^*∗∗∗*^*P* < 0.001; ^*∗∗∗∗*^*P* < 0.0001 by post hoc Tukey's one-way ANOVA statistical analysis.

The gene expression level of tight junctions including *occludin*, *ZO-1*, *claudin-2*, and adherens junction protein *E-cadherin* in response to 10 *µ*g/mL OMVs was investigated in Caco-2 cells and compared with the effect of live LF82 and DH5*α* as control strains (Figures 4(a)–4(d)). Based on the gene expression analysis, LF82-derived OMVs significantly decreased the expression of *occludin*, *ZO-1*, and *E-cadherin* (*P* < 0.0001), while increased *claudin-2* expression (*P* < 0.0001) in Caco-2 cells as compared to untreated cells. Also, OMV-treated cells indicated lower mRNA expression levels of *occludin*, *ZO-1*, and *E-cadherin* (*P* < 0.0001, *P* < 0.001, and *P* < 0.001, respectively) and higher expression of *claudin-2* (*P* < 0.001) compared to those cells treated with DH5*α*. No significant difference was seen between the impact of live LF82 and its derived OMVs on gene expression of *occludin*, *ZO-1*, and *E-cadherin* in Caco-2 cells; however, live LF82 induced a higher expression level of *claudin-2* than that of OMVs (*P* < 0.05). Nonetheless, there was a statistically significant difference between the impact of OMVs and LPS on the expression level of junction-associated genes in Caco-2 cells (*P* < 0.05). Furthermore, different cell treatments by OMVs and live bacteria (LF82 and DH5*α* strains) pretreated with polymyxin B were used to avoid the influence of LPS on gene expression of cell adhesion-related genes in Caco-2 cells. The results showed that polymyxin B pretreatment significantly affected the potential of OMVs and live LF82 to modulate the gene expression of *occludin*, *ZO-1*, *E-cadherin*, and *claudin-2* in Caco-2 cells.

### 3.5. OMVs Isolated from AIEC Strain LF82 Stimulated the Production of IL-8 and TNF-*α* in Caco-2 Cells

**Figure 5:**
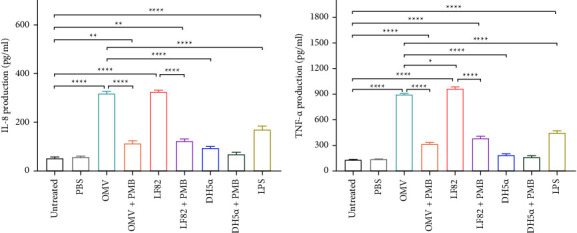
Quantification of cytokines (a) IL-8 and (b) TNF-*α* released by Caco-2 cells in response to AIEC OMVs, and the live strains of LF82 and DH5*α*. Data were shown as the mean ± SEM. ^*∗*^*P* < 0.05; ^*∗∗*^*P* < 0.01; ^*∗∗∗*^*P* < 0.001; ^*∗∗∗∗*^*P* < 0.0001 by post hoc Tukey's one-way ANOVA statistical analysis.

As shown in Figures 5(a) and 5(b), OMVs derived from the LF82 strain were able to significantly provoke the production of IL-8 and TNF-*α* in Caco-2 cells almost as same as live LF82 when compared to untreated control (*P* < 0.0001). In comparison with DH5*α*, OMVs remarkably induced a higher level of IL-8 and TNF-*α* production in Caco-2 cells (*P* < 0.0001). Moreover, the impact of OMVs on the induction of IL-8 and TNF-*α* by Caco-2 cells was more pronounced than those of LPS (*P* < 0.0001). Furthermore, pretreatment of OMVs and live bacteria (LF82 and DH5*α* strains) with polymyxin B was carried out to neutralize the contribution of LPS to cytokine production. Based on ELISA results, pretreatment with polymyxin B reduced the impact of live LF82 and its derived OMVs on the production of IL-8 and TNF-*α* cytokines.

## 4. Discussion

AIEC is a particular pathotype of *E. coli*, which was first isolated in 1998 from the ileal lesions of patients with CD [24]. Recent studies suggest the involvement of AIEC pathobionts in promoting intestinal inflammatory process by the activation of the gut immune system [8, 9, 25, 26]. The intestinal epithelial barrier acts as the first line of physical defense that separates commensal and pathogenic microbes in the lumen from underlying immune cells [27]. Epithelial cells are interconnected by the apical junctional complex, including tight junctions and adherens junctions, which are responsible for regulating epithelial permeability and integrity [28]. There is some evidence, suggesting that AIEC bacteria can disrupt the epithelial apical junctional complex, resulting in altered intestinal permeability [25].

The capacity of Gram-negative bacteria to secrete OMVs, which carry various components of the parent bacterium in a nonreplicative form, is widely acknowledged [12]. OMVs are known to contain a high proportion of PAMPs, including LPS, lipoproteins, lipoteichoic acid, and peptidoglycan, which can be identified by TLRs and other PRRs [12]. For instance, lipoproteins found in MVs released by mycobacterial species stimulate TLR-2, flagellins present on OMVs from EHEC O157 activate TLR-5, and nucleic acids associated with MVs from *S. aureus* or *Porphyromonas gingivalis* can activate TLR-7, TLR-8, and TLR-9 [29–32]. Notably, the LPS associated with OMVs from *E. coli* activate TLR-4 on the surface of host cells as well as intracellular TLR-4 [33]. In the case of OMVs derived from AIEC strain LF82, it has been demonstrated that they harbor outer membrane proteins such as OmpA, OmpC, and the maltose-binding protein MalE, which serves as a distinctive marker for periplasmic proteins [17]. Particularly, OmpA protein plays multiple roles in adhesion, invasion, and persistence of intracellular bacteria, and studies by Rulhion et al. have reported that OMVs, through the transmembrane protein OmpA, contribute to the invasion process of *E. coli* LF82 by enabling fusion with intestinal epithelial cells. This indicates that OMVs released by AIEC strains can facilitate host-microbe crosstalk through the transportation of bacterial effectors [16, 17]. Due to the composition of OMVs, these vesicles have been implicated in various physiological processes, including protein transport, nutrient acquisition, intercellular communication, antibacterial activity, toxin delivery, and induction of host immune responses. However, studies focusing on OMVs from AIEC LF82 and their specific inflammatory effects remain limited. Therefore, there is a crucial need to investigate the immunostimulatory potential and elucidate the underlying molecular mechanisms of these bacterial OMVs. In our study, we addressed this gap by specifically examining the proinflammatory activity of OMVs isolated from AIEC strain LF82. Our findings demonstrated their ability to robustly induce cytokine production in intestinal epithelial cells and consequently influence the gene expression of junction-associated proteins in the Caco-2 cell line.

Results obtained from a study by Schaar et al. demonstrated that TLR-2 is involved in the secretion of IL-8 in epithelial cells in response to OMVs from *Moraxella catarrhalis* [34]. In addition, Park et al. observed that inflammatory responses induced by OMVs from *Pseudomonas aeruginosa* are partly mediated by TLR-2 signaling [35]. In line with their findings, we found that OMVs derived from AIEC strain LF82 increased the gene expression of TLR-2 in Caco-2 cells, suggesting the presence of TLR-2 ligands within OMVs.

LPS as one of the most potent and immune-stimulating components of OMVs is considered a PAMP that can be sensed by TLR-4 expressed on human epithelial cells [26, 36]. Zhao et al. showed that OMVs isolated from *P. aeruginosa* can increase the expression level of TLR-4, MyD88, and NF-κB in human epithelial cells [37]. Another study by Behrouzi et al. also reported that the expression of TLR-4 was increased in Caco-2 cells upon treatment with OMVs released by enterotoxigenic *E. coli* [38]. In keeping with given expectation, our results demonstrated that OMVs purified from AIEC strain LF82 could trigger TLR-4 expression similar to live bacteria. We also observed a significant difference between the impact of OMVs and LPS on the gene expression of TLR-4, implying that OMVs may be more potent TLR-4 activators as compared to free LPS.

Polymyxin B is a cyclic cationic polypeptide antibiotic that neutralizes the LPS effect through binding to lipid A portion [39]. Ellis et al. have reported that LPS removal from OMVs by polymyxin B inhibited sensing via the TLR-4 complex, resulting in decreased response to the non-LPS components of vesicles [40]. In another study by Gowayed et al., it was shown that neutralization of LPS from OMVs content by polymyxin B inhibits TLR-2 and TLR-4 signaling pathways [39]. In accordance with their findings, we demonstrated that polymyxin B-pretreated OMVs showed a reduction in TLR-4 and TLR-2 expression.

TLR stimulation is linked to the activation of NF-*κ*B and other transcription factors leading to the upregulation of genes responsible for proinflammatory cytokines [36]. The potency of OMVs in induction of host inflammatory responses was evident in several bacteria, such as *Salmonella typhimurium* [41], *Pseudomonas aeruginosa* [42], and *Helicobacter pylori* [43]. Alaniz et al. also reported that OMVs isolated from *S. typhimurium* could activate macrophages and dendritic cells to secrete proinflammatory mediators TNF-*α* and IL-12 [41]. Moreover, another study by Bauman et al. showed that OMVs isolated from a variety of *P. aeruginosa* strains stimulated the secretion of IL-8 in lung epithelial cells [42]. Similarly, OMVs generated from *H. pylori* were found to elicit IL-8 production by gastric epithelial cells [43]. In agreement with these studies, our data indicated that OMVs from AIEC strain LF82 were potent stimulators of proinflammatory cytokines and induced the secretion of TNF-*α* and IL-8. This inflammatory response elicited by OMVs may be involved in the pathogenesis of AIEC through promoting the host immune dysregulation. The release of IL-8 into the supernatant of Caco-2 cells treated with OMVs was comparable to that of cells treated with live LF82, whereas live LF82 had a stronger effect on the production of TNF-*α* than that of OMVs. We also compared the effects of free LPS and OMVs on Caco-2 cells and found that OMVs could induce TNF-*α* and IL-8 secretion at higher levels than that of free LPS. In this regard, Park et al. demonstrated that OMVs derived from *P. aeruginosa* acted as more powerful inflammatory response inducers than that of LPS alone [35]. Besides, we demonstrated that LPS removal from OMVs by polymyxin B decreased both IL-8 and TNF-*α* secretion in Caco-2 cells; however, there was still a significant difference in cytokine production compared to untreated cells. In consonance with our results, Ellis et al. found that pretreatment of OMVs with polymyxin B reduced inflammatory cytokine production at both transcriptional and protein levels [40]. Taken together, our results indicate that both LPS and non-LPS components of OMVs are synergistically contributed to stimulate inflammatory response, suggesting that recognition of different PAMPs present on the OMVs through distinct TLRs possibly can promote additive effects.

It has been reported that pathogenic bacteria target apical junctional complex proteins via different ways such as direct contact, secretion of toxins or effectors, and indirect effects on host signaling pathways, which are implicated in the regulation of junctional proteins expression [44]. For instance, enterohemorrhagic *E. coli* O157:H7 disrupts the tight junction integrity through the downregulation of *ZO-1* and *occludin* [45]. Moreover, Muza-Moons et al. demonstrated that infection of intestinal epithelial cells with enteropathogenic *E. coli* caused aberrant distributions of *ZO-1*, *occludin*, and *claudin-1* [46]. AIEC strains have previously been shown to alter the function of intestinal epithelial barrier through displacing *ZO-1* and *E-cadherin* and increasing the expression of the pore-forming protein *claudin-2*, leading to facilitated bacterial translocation [47, 48]. Several studies have also shown that the production of proinflammatory cytokines can contribute to increased intestinal epithelial permeability by affecting the apical junctional complex [49, 50]. Mankertz et al. demonstrated that TNF-*α* treatment induced the expression of *claudin-2* in HT-29/B6 cells, leading to impaired intestinal epithelial integrity [51]. Our results showed that OMVs purified from AIEC strain LF82 altered the junction-associated proteins through downregulation of *occludin*, *ZO-1*, and *E-cadherin* and upregulation of *claudin-2* expression. As a matter of fact, the alteration in the expression of junctional proteins can, at least in part, be mediated through the production of proinflammatory cytokines provoked by OMVs. Furthermore, LPS of Gram-negative bacteria has been reported to alter the tight junction integrity and intestinal epithelial homeostasis [52]. Han et al. demonstrated that injection of *E. coli* O111:B4 LPS in C57Bl/6J mice decreased the expression of *ZO-1* and *occludin* in liver tissue [53]. Consistent with their finding, our results also showed a significant change in the gene expression of junction-associated proteins in Caco-2 cells treated with LPS. However, the impact of OMVs on the expression level of junctional proteins was more profound than those of LPS. Interestingly, we observed that polymyxin B pretreatment could reduce the potency of OMVs in disruption of the apical junctional complex. However, non-LPS components of vesicles still exhibited the capability to target junction-associated proteins but at a lower level. Taken together, these data suggest that the presence of various bioactive components in the cargo of OMVs may administer bacteria with the capability of inducing a more robust response.

## 5. Conclusion

In conclusion, our findings support the hypothesis that OMVs derived from AIEC strain LF82 are implicated in the secretion of proinflammatory cytokines. Furthermore, our results, for the first time, showed an overexpression of TLRs and junction-associated proteins in response to AIEC-derived OMVs in Caco-2 cells. These results once again support the notion that OMVs have the potential to induce host responses at a distance from the parent bacterium. However, there are some limitations in the present study. Firstly, the use of neoplastic Caco-2 cells as a model may not fully reflect the complex interactions that occur *in vivo*, and therefore, the findings should be generalized with caution. To gain a more precise understanding of the underpinning mechanisms by which OMVs isolated from AIEC strains stimulate the host inflammatory response and contribute to the pathogenesis of IBD, in-depth *in vivo* research is required. Moreover, further quantitation of the target genes at protein level is necessary to provide a more comprehensive understanding of molecular mechanisms underlying the observed changes and validate our gene expression results. Furthermore, studies are required to decipher the putative role of OMVs in the pathogenesis of AIEC pathobiont and its associated pathology.

## Figures and Tables

**Table 1:** The list of RT-qPCR primers used in this study.

Target gene	Oligonucleotide sequence (5′–3′)	Amplicon (bp)	References
*Occludin*	5′-CCAATGTCGAGGAGTGGG-3′	237	[18]
	5′-CGCTGCTGTAACGAGGCT-3′		

*ZO-1*	5′-CAAGATAGTTTGGCAGCAAGAGATG-3′	183	[18]
	5′-ATCAGGGACATTCAATAGCGTAGC-3′		

*Claudin-2*	5′-ACCTGCTACCGCCACTCTGT-3′	91	[19]
	5′-CTCCCTGGCCTGCATTATCTC-3′		

*E-cadherin*	5′-GAAGGTGACAGAGCCTCTGGAT-3′	122	[20]
	5′-GATCGGTTACCGTGATCAAAAT-3′		

TLR-2	5′-CAGAACTGCAGGTGCTGG-3′	163	[21]
	5′-ACACCTCTGTAGGTCACTGTTG-3′		

TLR-4	5′-CAGAACTGCAGGTGCTGG-3′	197	[22]
	5′-GTTCTCTAGAGATGCTAG-3′		

*β*-*actin*	5′-TCACCCACACTGTGCCCATCTACGA-3′	295	[23]
	5′-CAGCGGAACCGCTCATTGCCAATGG-3′		

## Data Availability

Data sharing is not applicable to this article as no datasets were generated or analyzed during the current study.
